# Contaminants of emerging concern in urine: a review of analytical methods for determining diisocyanates, benzotriazoles, benzothiazoles, 4-methylbenzylidene camphor, isothiazolinones, fragrances, and non-phthalate plasticizers

**DOI:** 10.1007/s11356-023-29070-y

**Published:** 2023-08-19

**Authors:** Žiga Tkalec, Agneta Annika Runkel, Tina Kosjek, Milena Horvat, Ester Heath

**Affiliations:** 1 Department of Environmental Sciences (O2), Jožef Stefan Institute, Jamova cesta 39, 1000 Ljubljana, Slovenia; 2 Jožef Stefan International Postgraduate School, Jamova cesta 39, 1000 Ljubljana, Slovenia

**Keywords:** Human biomonitoring, Exposure, Exposome, Sample preparation, Biomarker, Mass spectrometry

## Abstract

Human biomonitoring (HBM) frameworks assess human exposure to hazardous chemicals. In this review, we discuss and summarize sample preparation procedures and analytical methodology for six groups of chemicals of emerging concern (CECs), namely diisocyanates, benzotriazoles, benzothiazoles, 4-methylbenzylidene camphor, isothiazolinones, fragrances, and non-phthalate plasticizers, which are increasingly detected in urine, however, are not yet widely included in HBM schemes, despite posing a risk to human health. The sample preparation procedures depend largely on the chemical group; however, solid-phase extraction (SPE) is most often used due to the minimized sample handling, lower sample volume, and generally achieving lower limits of quantification (LOQs) compared to other extraction techniques. In terms of sample analysis, LC-based methods generally achieve lower limits of quantification (LOQs) compared to GC-based methods for the selected six groups of chemicals owing to their broader chemical coverage. In conclusion, since these chemicals are expected to be more frequently included in future HBM studies, it becomes evident that there is a pressing need for rigorous quality assurance programs to ensure better comparability of data. These programs should include the reporting of measurement uncertainty and facilitate inter-laboratory comparisons among the reporting laboratories. In addition, high-resolution mass spectrometry should be more commonly employed to enhance the specificity and selectivity of the applied analytical methodology since it is underrepresented in HBM. Furthermore, due to the scarcity of data on the levels of these CECs in urine, large population HBM studies are necessary to gain a deeper understanding of the associated risks.

## Introduction

With the emergence of new consumer and personal care products, the gradual awareness of potentially hazardous chemicals entering the environment has also increased, especially when combined with improved analytical methods and instrumentation, such as chromatographic and high-resolution mass spectrometric techniques that have allowed the detection of previously overlooked contaminants. Additionally, knowledge of the toxic effects of known compounds is increasing, many of which are now considered contaminants of emerging concern (CECs). However, human exposure to many compounds and even groups of compounds remains under-researched, especially in routine monitoring schemes, such as the National Health and Nutrition Examination Survey (NHANES), Human Biomonitoring for Europe (HBM4EU), and the German Environmental Survey (GerES) (Yadav et al. 2021).

Human biomonitoring (HBM) measures potentially harmful chemicals and their metabolites in human samples to identify exposure sources, understand chemical risk, and ensure policy informing and efficiency of reduction strategies (Ganzleben et al. 2017). As such, it measures internal exposure that reflects the actual chemical uptake from oral, dermal, and inhalation exposure pathways (Vorkamp et al. 2021). Blood and urine are the most commonly investigated matrices. Although blood represents an optimal matrix since it is in contact with all tissues in the body and allows the partitioning of compounds into every organ, sampling blood is invasive which makes it less suitable for specific populations such as children and infants. In contrast, urine can be non-invasively collected as a 24-h composite or, more commonly, as a spot sample. The main disadvantage of collecting urine is the varying degrees of dilution due to an individual’s hydration status, albeit adjustment methods like normalization to creatinine, specific gravity, and osmolarity can circumvent this issue (Esteban and Castaño 2009).

Although HBM schemes already include many chemicals, groups of potentially harmful compounds remain that have not yet been adequately addressed. These include, for example, pyrrolidones, isocyanates, benzotriazoles, benzothiazoles, ultra-violet (UV) filters, antimicrobials (MCI, MI), fragrances, and non-phthalate plasticizers, where evidence for their adverse health effects is mounting (Kolossa-Gehring et al. 2017; Salthammer 2020). In this paper, we review the analytical methods quantifying these compounds in human urine to compile data that could be used for future HBM studies.

## Groups of under-researched CECs

**Isocyanates **are raw materials used to manufacture polyurethane foams, adhesives, and paints (Lépine et al. 2020). The most common isocyanates are methylene diphenyl diisocyanate (MDI), toluene 2,4 diisocyanate (4TDI), toluene 2,6 diisocyanate (6TDI), naphthylen-1,5-diisocyanate (NDI), 1,4-phenylene diisocyanate (PPDI), and 1,6-hexamethylene diisocyanate (HDI). Once in the body, isocyanates are hydrolysed to their corresponding amines, namely methylene diphenyldianiline (MDA), 2,4-toluenediamine (4TDA), 2,6-toluenediamine (6TDA), 1,5-naphthalenediamine (NDA), p-phenylenediamine (PPDA), and hexamethylenediamine (HDA), which are used as biomarkers of exposure. Due to their high reactivity, they can form protein adducts that, after protein degradation, are excreted in urine as conjugates, particularly with glutathione (Sabbioni et al. 2010). Several acute and chronic effects connected to isocyanate exposure have been reported, such as irritation of the eyes, skin, mucous membranes, and respiratory system, asthma, and reduced lung function (Lépine et al. 2020). A study of the long-term effects of one-time high-dose exposure reported the occurrence of diseases such as diabetes, hypertension, cancer, reproductive outcomes, and respiratory/orthopaedic/general morbidity in the exposed population (Ganguly et al. 2018).

**Benzotriazoles** (BTRs) are high production volume chemicals used as corrosion inhibitors and UV stabilizers for various photography and liquid coolant applications. Benzothiazoles (BTHs) are also used as corrosion inhibitors, industrial biocides, and vulcanisation accelerators in rubber production (Asimakopoulos et al. 2013b; Maceira et al. 2018; Naccarato et al. 2014). In the human body, they are biotransformed into more hydrophilic metabolites that are excreted as glucuronidated and sulphated compounds (Asimakopoulos et al. 2013b). Although data on their toxicological effects are limited, available evidence suggests that they can adversely affect the liver and kidney (Naccarato et al. 2014), while BTH is a suspected carcinogen (Li et al. 2018b).

**Pyrrolidones** (*N*-methylpyrrolidone-NMP and *N*-ethylpyrrolidone-NEP) are aprotic polar solvents used in the petrochemical and microelectronics industries for producing dyes, biocides and cosmetics (Schmied-Tobies et al., 2021). Due to their volatility, the main route of exposure is inhalation, although ingestion and dermal absorption have also been reported (Suzuki et al. 2009). Their main metabolites and biomarkers of exposure are 5-hydroxy-*N*-methyl-2-pyrrolidone (5-HNMP), 2-hydroxy-*N*-methylsuccinime (2-NMSI), 5-hydroxy-*N*-ethyl-2-pyrrolidone (5-HNEP), and 2-hydroxy-*N*-ethylsuccinimine (2-HESI). Pyrrolidones are skin, eye, and respiratory tract irritants and exhibit reproductive effects (Kirman 2020; Kirman et al. 2023).

Since phthalate-based plasticizers have been under criticism due to their adverse health effects, **phthalate alternatives** have entered the market with a constantly increasing share (Bui et al. 2016). Phthalate alternatives find applications as softeners in plastics and additives in personal care products and industrial materials. Although many have been well-studied, e.g. diisononyl ester of cyclohexane-1,2-dicarboxylic acid (DINCH), less is known about tri-(2-ethylhexyl) trimellitate (TEHTM), di-2-ethylhexyl adipate (DEHA), di-*n*-butyl adipate (DnBA), and di-(2-ethylhexyl) terephthalate (DEHTP). Most of these are alternatives for the traditional phthalate plasticizer di-2-ethylhexyl phthalate (DEHP), while DnBA is an alternative for di-*n*-butyl phthalate (DnBP). Their toxicity and migration rates are significantly lower than traditional phthalates; however, leaching into the surrounding media still occurs; for example, DnBA is restricted to low temperature applications due to high migration rates. This compound is often used in high concentrations (up to 8% of the final product) (Andersen 2006) in personal care products, solvents, and cleaning products. It exhibits very low toxicity in humans with a no-observed-adverse-effect level of 300 mg/kg bw (Ringbeck et al. 2020). However, estimating human exposure to DnBA is a challenge due to the lack of exposure data, intake rate estimates, and data on health effects. For instance, there is currently no data on the potential endocrine disruptive potential of DnBA (Bui et al. 2016). Monitoring is therefore needed as a preventive measure due to the increasing exposure of the population (Bui et al. 2016).

**DEHA** is a high production volume adipate, and human exposure is continuously increasing (Bui et al. 2016). Animal studies show evidence of adverse health outcomes in rats following exposure to high doses of DEHA (Silva et al. 2013). **DEHTP** is a structural isomer of DEHP that has not shown any of the reported adverse health effects of DEHP, with a reported NOEL of 500 mg/kg bw/day. It is produced in quantities similar to DINCH in the European Union (> 10,000 t/year) and has been frequently detected in household dust and indoor air and humans are widely exposed to this compound (Bui et al. 2016). **TEHTM** is a tri-ester structurally similar to DEHP but has lower toxicity. It is, however, classified as a Category 2 reproductive toxicant, a Category 3 specific target organ toxicant, and is relatively environmentally persistent (half-life 16–60 days). As such, TEHTM has a set derived no effect level of 1.13 mg/kg bw/day within the European Chemicals Agency (ECHA) registration (Höllerer et al. 2018a).

These phthalate alternatives fall under the high molecular–weight compounds (> 6 C atoms in the side chain) and undergo similar metabolic pathways in humans. The tri-ester compound TEHTM can form three diester isomers during hydrolysis, followed by further hydrolysis to monoesters. TEHTM, DEHA, DnBA, and DEHTP also undergo biotransformation to secondary metabolites containing hydroxy, oxo or carboxy groups on the side chain. We included them in the list of phthalate alternatives due to their expected increasing market share, limited exposure data and limited toxicological data.

**Organic ultra-violet (UV) filters** are a group of compounds capable of filtering UV radiation due to their degree of conjugation and are used in personal care products such as sunscreens and other cosmetic products to protect the skin. They can be roughly classified into benzophenone (BP), cinnamate, crylene, camphor and salicylate derivatives. The latter consists of other organic UV filters that do not fall into the abovementioned groups (Huang et al. 2021). For example, although organic UV filters are highly studied in HBM, an exception is the camphor derivative 4-methylbenzylidene camphor (MBC). MBC is currently detected at relatively low concentrations (<LOD — 13.93 ng/mL) and frequencies, with most studies focusing on aquatic systems, where it is known to be highly lipophilic and accumulates in the fatty tissue of biota (Ao et al. 2018b; Huang et al. 2021). The metabolic pathway of MBC in humans has been studied after dermal application. 3-(4′-carboxybenzylidene)camphor (CBC) and four isomers of 3-(4′-carboxybenzylidene)hydroxycamphor (CBC-OH) were identified as the main metabolites of MBC and are the main biomarkers of exposure (León-González et al. 2013).

Human exposure to MBC can result from either direct dermal application or ingestion of contaminated food and drinks, for instance, fish consumption or tap water (Li et al. 2019). Health concerns associated with MBC have increased due to its suspected endocrine activity, while in vitro studies suggest that MBC can enhance the process of apoptosis and hence directly affect nerve cells (Broniowska et al. 2016). Other studies report adverse effects on the immune system (Ao et al. 2018a), and it has been suggested that MBC facilitates the migration of human breast cancer cells (Alamer and Darbre 2018). Studies investigating the toxicological profile of MCB report mild endocrine disruptive effects on the thyroid gland and the potential to delay tissue growth and placenta formation during early pregnancy (HBM4EU 2020)

Exposure to **fragrances** is widespread as they find applications in personal care products and scented non-cosmetic products. The main pathways of exposure are inhalation and dermal absorption. The most common health endpoint of fragrances is an allergic response, with some studies reporting endocrine disruptive effects (Dodson et al., 2012). Musk xylene (MX) and musk ketone (MK) are nitro musks, which have now been replaced in most countries due to their estrogenic activity and neural toxicity (Taylor et al., 2014) with polycyclic musks, such as celestolide (ADBI), traseolide (ATII), galaxolide (HHCB), and tonalide (AHTN). MX is classified under REACH as a chemical of high concern, while in the case of MK, a warning about its use has been placed (Taylor et al. 2014). Polycyclic musks are under scrutiny because of their persistence and adverse health effects, such as estrogenic activity. Along with musks, other synthetic fragrances of concern include lysmeral with the known metabolites lysmerol, lysmerylic acid, hydroxylysmerylic acid, tert-butyl hippuric acid, (TBHA), and tert-butyl benzoic acid (TBBA) (Scherer et al., 2017) and 7-hydroxycitronellal (7-HC) with the main metabolite 7-hydroxycitronellic acid (7-HCA). Known health endpoints of 7-HC are skin irritation and skin sensitization (Stoeckelhuber et al. 2018), whereas endocrine disruptive effects are suspected (Scherer et al. 2021).

Frequently used **antimicrobials** in personal care products are isothiazole and its derivatives. The most extensively used among them are based on isothiazolinone. The knowledge of their potential adverse health effects has been emerging only recently. Methylchloroisothiazolinone (MCI) and methylisothiazolinone (MI) are often used to enhance the overall antimicrobial effect and find application in preserving solutions in personal care products and industrial products. Human exposure is mainly the result of dermal applications. Both MCI and MI undergo rapid biotransformation in the human body with a mean half-life of 3.6 h. The main biomarker of MCI/MI exposure is *N*-methylmalonamic acid (NMMA), whereas acetylamino([3-(methylamino)-1-(methylthio)-3-oxopropyl]thio)acetic acid (M-12) is a minor metabolite with an excretion fraction of between 10 and 23% in humans (Schettgen et al. 2021b). MCI/MI are broad-spectrum antimicrobials with high efficiency at trace concentrations (Silva et al. 2020). Over recent years, concerns over their sensitization potential and allergic dermatitis and other adverse health outcomes such as cytotoxicity have increased, leading to an interest in monitoring these compounds (Castanedo-Tardana and Zug 2013; Park and Seong 2020). MCI has a higher potential for the mentioned health endpoints (Silva et al. 2020).

## Occurrence of selected CECs in urine

Most of the publications included in this review verified their respective method by implementation in HBM studies of varying scales, thereby confirming the occurrence of CECs in human urine (Table 1). Most of the studied CECs (fragrances, BTR and BTH, antimicrobials, pyrrolidones, and diisocyanates) are frequently detected in urine, whereas the UV filter MBC was rarely detected, and if detected at all, it was in trace amounts (Ao et al. 2018b; Frederiksen et al. 2017; Leng and Gries 2017). The included non-phthalate plasticizers, on the other hand, are frequently detected and in relatively high concentrations, with a maximum concentration of 1165 ng/mL of OH-MEHTP (Bastiaensen et al. 2020). Therefore, it is clear that the presumably non-exposed general population is frequently exposed to most of the included CECs and highlights the need to include these compounds in routine HBM studies.

**Table 1:** Occurrence of CECs in urine

Compound group	Analyte	Median (ng/mL)	Mean (ng/mL)	Range (ng/mL)	population	Reference
Fragrances	ADBI			n.d.		Chen et al. (2018)
	AHMI			1.38–2.65		
	ATII			n.d.		
	HHCB			0.48–2.09		
	AHTN			0.14–0.57		
	MX			n.d.		
	MK			n.d.–1.49		
	Lysmerol	0.8	2.25 ±6.14	0.1–38.7		Pluym et al. (2016)*
	Lysmerylic acid	0.8	1.25 ± 1.59	0.02–9.2		
	TBBA	19.6	28.7 ± 34.3	5.3–188.9		
	OH-lysmerylic acid	0.06	0.76 ± 1.46	0.01–8.2		
	7-HCA	15.1	34.4 ± 85.2	4.9–557.0		Stoeckelhuber et al. (2017)*
BTRs & BTHs	1H-BTR		1.23	<LLOQ–5.67	males	Asimakopoulos et al. (2013a)*
			1.63	0.85−3.16	females	
	1-OH-BTR		–	–	males	
			–	–	females	
	TTR		0.70	<LLOQ–2.14	males	
			0.72	<LLOQ−3.37	females	
	XTR		0.39	<LLOQ–2.39	males	
			–	3.69	females	
	5-Cl-1H-BTR		–	–	males	
			–	–	females	
	BTH		3.51	<LLOQ–16.7	males	
			5.59	<LLOQ−34.3	females	
	2-OH-BTH		1.55	<LLOQ	males	
			2.40	<LLOQ−6.00	females	
	2-Me-S-BTH		–	–	males	
			–	–	females	
	2-amino-BTH		0.11	<LLOQ–0.30	males	
			0.17	<LLOQ−0.25	females	
	2-SCNMeS-BTH		–	–	males	
			–	–	females	
	MTB (total)	< 1	< 1	< 1–10.8	Non-exposed	Gries et al. (2015)
		4527	3958	567–6210	exposed	
	MTB (free)	70	69	< 1–137	exposed	
	1-OH-BTR	0.78	1.13	<LOQ-1.13		Li et al. (2017)
	1H-BTR	2.45	2.4	<LOQ-4.12		
	TTR	2.38	2.46	<LOQ-4.79		
	5-Cl-BTR	n.d.	n.d.	n.d.		
	XTR	2.38	2.46	<LOQ–4.79		
	BTH	–	–	1.74		
	2-OH-BTH	–	–	0.38		
	2-amino-BTH	0.26	0.28	<LOQ–0.68		
	2-MeS-BTH	n.d.	n.d.	n.d.		
	2-SCNMeS-BTH	n.d.	n.d.	n.d.		
	1H-BTR	1.09	1.31	<LOD–3.86	Pregnant women	Li et al. (2018b)
	1-OH-BTR	0.21	0.25	<LOD–1.39		
	TTR	0.22	0.27	<LOD–0.90		
	5-Cl-1H-BTR	0.015	0.023	<LOD–0.096		
	BTH	1.13	1.49	0.016–3.91		
	2-OH-BTH	<LOD	<LOD	<LOD		
	1-H-BTR		0.10	?–36		Zhou et al. (2018)#
	1-OH-BTR		0.32	?–330		
	XTR		0.04	?–18		
	TTR		0.036	?–6.1		
	BTH		1.5	?–53		
	2-OH-BTH		0.28	?–160		
	2-MeS-BTH		0.30	?–15		
	2-NH_2_-BTH		0.017	?–8.3		
	2-SCNMeS-BTH		<LOD	?–0.66		
	5-Cl-H-BTR		<LOD	?–0.53		
Antimicrobials	NMMA	3.6		?–7.5	Non-exposed males	Schettgen et al. (2017)*
		2.9		?–11.8	Non-exposed females	
	M-12	0.57*		0.20–1.79*	Non-exposed	Schettgen et al. (2021b)
	NMMA	2.7		0.9–9.6		
Diisocyanates	MDA			0.1–0.2*		Henriks-Eckerman et al. (2015)
	MDA		3.30	2.0–4.0	Factory workers	Mirmohammadi et al. (2013)*
	HDA			0.06–5.96		Robbins et al. (2018)
	TAHI			<LOD–1.99		
	MDA			<LOD–15.18		Sun et al. (2018)
	6TDA			<LOD–8.46		
	4TDA			<LOD–2.35		
Pyrrolidones	5-HNMP	0.39			exposed	Haufroid et al. (2014)*
	2-HMSI	0.56				
	5-HNMP	0.09			Non-exposed	
	2-HMSI	0.23				
	5-HNMP	69.5		?–620.0	Non-exposed	Schindler et al. (2012)
	2-HMSI	63.5		?–256.2		
	5-HNEP	< 15.0		?–769.3		
	2-HESI	< 5.0		?–310.8		
UV filters	MBC	2.46	0.65	<LOD–13.93		Ao et al. (2018b)*
	MBC	–		<LOD	Children/adolescents	Frederiksen et al. (2017)
	CBC			<LOD	Non-exposed	Leng and Gries (2017)
	CBC-OH			<LOD		
Non-phthalate plasticizers	1-MEHTM	0.10	0.52	<LOQ–13.1		Pinguet et al. (2019)
	2-MEHTM	0.47	25.6	<LOQ–925		
	4-MEHTM	0.04	0.19	<LOQ–4.14		
	1-MEHTM		455			Höllerer et al. (2018b)
	2-MEHTM		1629			
	4-MEHTM		16.0			
	5OH-1-MEHTM		308			
	2OH-2-MEHTM		256			
	5oxo-1-MEHTM		81.4			
	5oxo-2-MEHTM		60.7			
	5cx-1-MEPTM		338			
	5cx-2-MEPTM		46.6			
	2cx-2-MMHTM		<LOD			
	2cx-1-MMHTM		14.6			
	1-DEHTM	<LOQ		<LOQ–0.89		Bastiaensen et al. (2020)
	2-DEHTM	<LOQ		<LOQ–0.91		
	MEHA	0.08	0.38	<LOQ–7.92		Pinguet et al. (2019)
	5OH-MEHA	<LOQ		<LOQ–0.07	Pregnant women	Nehring et al. (2019)
	5oxo-MEHA	<LOQ		<LOQ–0.13		
	5cx-MEHA	<LOQ		<LOQ–0.24		
	5OH-MEHA	<LOQ		<LOQ	adults	
	5oxo-MEHA	<LOQ		<LOQ		
	5cx-MEHA	<LOQ		<LOQ–0.24		
	MEHA	0.02		0.02–3.18		Bastiaensen et al. (2020)
	OH-MEHA	0.92		0.07–2.94		
	MnBA	<LOQ		?–0.18		Ringbeck et al. (2020)
	3OH-MnBA	<LOQ		<LOQ		
	3cx-MnBA	2.54		?–78.3		
	MEHTP	<LOQ	0.21	<LOQ–9.90		Pinguet et al. (2019)
	MEHTP	0.02		0.02–50.8		Bastiaensen et al. (2020)
	OH-MEHTP	0.51		0.07–1165		

*Creatinine adjustment

#SG adjustment

## Sample preparation and analytical methods for the determination of CECs in urine

Once in the human body, many compounds undergo phase I and II metabolism involving esterases, lipases, and enzymes of the cytochrome P450 family (phase I) (Iyanagi 2007). Phase II metabolism occurs mainly in the liver, although these enzymes are expressed to a lower degree also in other tissues such as kidneys and intestine. The main phase II reactions are glucuronidation, sulfation, and glutathione conjugation (James 2021). In order to measure total CECs (free and conjugated), enzymatic deconjugation is usually applied, using enzymes like β-glucuronidase/arylsulfatase or acid/base hydrolysis (Glauser et al. 2014).

Sample clean-up and extraction procedures are needed to remove unwanted matrix constituents, reduce matrix effects, and eliminate interferences. Furthermore, during extraction, the sample is usually concentrated to detect low levels of biomarkers of exposure at concentrations significant for HBM. Solid phase extraction (SPE) is a commonly used method for preparing biological samples (Bocato et al. 2019). Compared to liquid-liquid extraction (LLE), it offers higher selectivity due to variously functionalized sorbents for the retention of analytes of choice with a wide range of physicochemical properties. The availability of SPE in 96-well format offers an adaptation to high-throughput sample preparation workflows, which is beneficial in HBM since large cohorts are analysed. However, it requires extensive parameter optimization.

Along with common extraction methods, other more specific extraction methods have been utilized, such as solid-supported liquid-liquid extraction (SSLE) on microporous diatomite cartridges to extract fragrances (Liu et al. 2015), ultrasound-assisted emulsification microextraction (USAEME) with tetrachloromethane for the extraction of polycyclic and nitro musks (Chen et al. 2018), and solid-phase microextraction (SPME) for extraction of BTRs and BTHs using polyacrylate fibre (Naccarato et al. 2014).

After extraction, which often includes a preconcentration step, the most commonly applied separation methods include liquid chromatography (LC) or gas chromatography (GC), but other approaches have been also used (see below). Due to the high degree of molecular functionalization and suitable polarity, most analysts use reversed-phase LC. High and ultra-performance LC (HPLC and UPLC) do not require high volatility and thermal stability of analytes and are suitable for analysing polar matrices, such as urine. For volatile analytes like fragrances, GC is the method of choice. GC can also separate semi- and non-volatile compounds and is often accompanied by an appropriate derivatization step, such as silylation.

Regarding analysis, separation by LC and GC is most commonly combined with mass spectrometry (MS) analysers. MS enables increased sensitivity and selectivity, particularly when using tandem MS (MS2). Compared to MS, which is performed as a full scan or by following a single compound-specific ion (selected ion monitoring, SIM), MS2 follows multiple fragmentations (multiple reaction monitoring, MRM), which offers higher selectivity and specificity, which is especially important in the analysis of complex samples like urine. This specificity is reflected in the methods reported here, as CECs were detected almost exclusively in MRM mode. Besides MS, other approaches are suitable for detecting certain compounds. In one case, HPLC with fluorescence detection was used to detect biomarkers of exposure of diisocyanates (Sun et al. 2018). This required derivatization using 4-(1-pyrene)butanoyl chloride (PBC) (Sun et al. 2018). Compared to the methods using negative chemical ionization (NCI) set-ups, such as GC NCI-SIM (Mirmohammadi et al. 2013), UPLC (Bhandari et al. 2016; Lépine et al. 2020, 2019a), and nano-UPLC with MRM (Robbins et al. 2018) were described for the detection of MDA. This approach yields ten times lower LOQ (0.001 ng/mL) and does require any complex sample preparation procedures (offline-SPE).

The suitability of the methods was tested following validation procedures, typically following validation guidelines, for example, Eurachem (Eurachem 2002) or the FDA Guidelines for Bioanalytical method validation (FDA 2001). As validation parameters, linearity, specificity, recovery, precision, and accuracy were tested. Precision and accuracy were always limited to at least 20%, but more often more stringent margins were used such as 15% (Asimakopoulos et al. 2013a; Stoeckelhuber et al. 2017) or even 10% (Bhandari et al. 2019; Höllerer et al. 2018b; Schettgen et al. 2017). Lépine et al. assessed the method’s accuracy by comparing the results with the samples from an external quality control assessment, such as the German External Quality Assessment Scheme (G-EQUAS). In most studies, the method’s LOD and LOQ were determined based on the signal-to-noise ratio, namely 3:1 and 10:1 signal-to-noise ratios for LOD and LOQ, respectively. An assessment based on 3- and 9-times standard deviation of the lowest limit of quantification (LLOQ) divided by the slope of the calibration curve was used in five studies (Asimakopoulos et al. 2013a; Höllerer et al. 2018a, 2018b; León-González et al. 2013; Pinguet et al. 2019). The measurement uncertainty was assessed only once in a study published by Chen et al. (Chen et al. 2018).

### Fragrances

For most fragrances, no deconjugation was performed before sample extraction and clean-up. The majority of nitro and polycyclic musks lack the functional groups for conjugation, whereas, for lysmerol, deconjugation was achieved enzymatically using β-glucuronidase. The reviewed methods for extracting fragrances from urine adopt an LLE approach using between 0.8 and 1 mL of sample volume.

Due to the inherent volatility of fragrance compounds, they were mainly analysed using GC methods, except for lysmeral and 7-HCA, where both GC and LC-based methods have been applied. A derivatization step is required for these two compounds to improve their volatility, whereas polycyclic and nitro musks are sufficiently volatile to be analysed via GC without further adaptations. The two methods for detecting polycyclic musks in urine (Chen et al. 2018; Liu et al. 2015) yielded comparable results in terms of LOQ. However, regarding LOQs, the method of Liu et al. (2015) using an SSLE approach yields slightly better results (Table 2) compared to using USAEME, as suggested by Chen et al. (2018).

**Table 2:** Sample preparation procedures and analytical methods for the selected CECs

Analyte family	Compounds	BoE	Sample preparation	Analysis		LOQ (ng/mL)	Ref.
				Separation	MS		
Fragrances	Polycyclic musks: ADBI, ATII, HHCB, AHTN	ADBI, ATII, HHCB, AHTN	SSLE: 0.8 mL sample loaded on a cartridge, equilibrated 10 min, extracted with 8 mL HEX. Dried under N_2_ and reconstituted in 80 μL of HEX	GC: TG-5HT column (15 m × 0.25 mm × 0.1 μm), program: 70 °C 5 min, 70–190 °C at 30 °C/min, 190 °C 3 min, 190–300 °C at 30 °C/min, 300 °C 3 min	EI-MS2-MRM: TSQ Quantum (Thermo Fischer), EI 40 eV	0.107, 0.143, 0.250, 0.017, 0.103	Liu et al. (2015)
	Polycyclic musks and nitro musks: ADBI, AHMI, ATII, HHCB, AHTN, MX, MK	ADBI, AHMI, ATII, HHCB, AHTN, MX, MK	USAEME: 1 mL mixed with 0.1 g NaCl and 50 μL of CCl_4_, ultrasonicated for 1 min at 40 °C. Then centrifuged at 7000 rpm for 3 min. Extract collected and 10 μL analysed	GC: Instrument Varian 450 (Walnut Creek, CA, USA), column: DB-5MS (30 m × 0.25 mm × 1.0 μm), Injection: 10 μL, 80 °C for 2 min then 200 °C/min to 280 °C Program: 70 °C, 4 min, 70–190 °C, 30 °C/min, 190–196 °C, 1 °C/min, 196–280 °C, 120 °C/min, 120 °C, 2.3 min	EI-MS2-MRM: Varian 220 (Walnut Creek, CA, USA)	0.25, 0.1, 0.5, 0.1, 0.1, 0.1, 0.25	Chen et al. (2018)
	Lysmeral	TBBA, lysmerol, lysmerylic acid, hydroxy lysmerylic acid, TBHA	LLE: 1 mL of deconjugated urine (37 °C, 16 h) acidified and extracted with DCM. Dried and derivatized with 3-NPA in WA (80 °C, 30 min)	UPLC: Waters Acquity UPLC I-Class Column: Waters Acquity BEH C18 (100 mm × 2.1 mm, 1.7 μm) Mobile phases: A (MeOH), B (5 mM ammonium acetate with 0.025% ammonium hydroxide, pH 9.2), flow 0.35 mL/min Gradient elution: 80% B, 0–4 min, 80–55% B, 4–10 min, 55–35% B, 10–11 min, 35–0% B, 11–11.1 min, 0% B, 11.1–13 min, 0–80% B, 13–13.1 min, 80% B, 13.1–15 min	ESI(-)-MS2-MRM: Waters TQ-S Triple Quadrupole	0.42, 0.1, 0.36, 0.45, 0.39	Pluym et al. (2016)
	7-HC	7-HCA	LLE: 1 mL urine deconjugated (37 °C, 3 h), acidified and extracted with DCM. Dried and derivatized with 3-NPA (80 °C, 30 min)	UPLC: Waters Acquity UPLC I-Class Column: Waters Acquity BEH C18 (100 mm × 2.1 mm, 1.7 μm) Mobile phases: A (0.1% FA/W), B (0.1% FA/ACN), flow 0.5 mL/min Gradient elution: 80% A, 0–1 min, 80–20% a, 1–5 min, 20% A, 5–5.5 min, 20–80% A, 5.5–7 min	ESI(-)-MS2-MRM: Waters TQ-S Triple Quadrupole	0.5	Stoeckelhuber et al. (2017)
Benzotriazoles and benzothiazoles	1H-BTR , 1OH-BTR, TTR, XTR, 5-Cl-1H-BTR, BTH, 2-OH-BTH, 2-MeS-BTH, 2-amino-BTH, 2-SH-BTH, 2-SCNMeS-BTH	1H-BTR , 1OH-BTR, TTR, XTR, 5-Cl-1H-BTR, BTH, 2-OH-BTH, 2-MeS-BTH, 2-amino-BTH, 2-SH-BTH, 2-SCNMeS-BTH	SPE: 2 mL, deconjugation with β-glucuronidase (37 °C, 24 h), diluted, pH adjusted to 3, extracted on Oasis HLB 6 cc/200 mg with MeOH/ACN (1:1), dried and reconstituted in 200 μL MeOH/ACN (1:1) LLE: 600 μL extracted with 6 mL ACN/DCM (1:1), extract dried and reconstituted in 150 μL MeOH/ACN	HPLC: Agilent 1100 HPLC Column: Zorbax SB aq (150 × 2.1 mm, 3,5 μm), Mobile phases: A (0.1% FA/W), B (ACN), flow 0.25 mL/min Gradient elution: 10–40% B, 4.5 min, 40–100% B, 11.5 min, 100% B, 6.1 min, 10% B, 7.1 min	ESI(+)-MS2-MRM:Applied Biosystems triple quadrupole	SPE: 0.50, 2.00, 0.20, 0.20, 0.50, 5.00, 2.50, 0.20, 0.20, 0.50 LLE: 1.70, 0.70, 0.70, 1.70, 0.70. 17.00, 3.50, 1.70, 0.70, 1.70	Asimakopoulos et al. (2013a)
	1H-BTR , 1OH-BTR, TTR, XTR, 5-Cl-1H-BTR, BTH, 2-OH-BTH, 2-MeS-BTH, 2-amino-BTH, 2-SCNMeS-BTH	1H-BTR , 1OH-BTR, TTR, XTR, 5-Cl-1H-BTR, BTH, 2-OH-BTH, 2-MeS-BTH, 2-amino-BTH, 2-SCNMeS-BTH	LLE: 1 mL deconjugated with β-glucuronidase (37 °C, 24 h), extracted with 3 mL MTBE/EA (5/1), dried and reconstituted in 200 μL ACN/W (6/4)	UPLC: Dionex Ultimate 3000 UHPLC Column: Thermo Hypersil GOLD (100 × 2.1 mm, 1.9 μm) Mobile phases: A (0.01% FA/W), B (ACN), flow 0.25 mL/min Gradient elution: 10–83% B, 9.5 min, 83–100%, 100% B, 3 min, 10% B	ESI(+)-MS2-MRM: Thermo Scientific TSQ Quantiva Triple Quadrupole	0.2, 11, 2, 7, 4, 9, 6, 11, 4, 14	Li et al. (2017)
	1H-BTR , 1OH-BTR, TTR, XTR, 5-Cl-1H-BTR, BTH, 2-OH-BTH, 2-MeS-BTH, 2-amino-BTH, 2-SCNMeS-BTH	1H-BTR , 1OH-BTR, TTR, XTR, 5-Cl-1H-BTR, BTH, 2-OH-BTH, 2-MeS-BTH, 2-amino-BTH, 2-SCNMeS-BTH	LLE: 1 mL deconjugated with β-glucuronidase (37 °C, 24 h), extracted with 3 mL MTBE/EA (5/1), repeated twice, dried and reconstituted in 200 μL ACN/W (6/4)	UPLC: Waters Column: Acquity BEH C18 (100 × 2.1 mm, 1.7 μm) Mobile phases: A (0.01% FA/W), B (ACN), flow 0.25 mL/min Gradient elution: 10% B, 0.5 min, 10% B, 9.5 min, 10–83% B, 2 min, 100% B, 2.8 min, 10% B	ESI(+)-MS2-MSM: Waters Xevo TQ-XS Triple Quadrupole	0.03, 0.12, 0.06, 0.03, 0.03, 0.06, 0.03, 0.06, 0.03, 0.24	Zhou et al. (2018)
	1H-BTR , 1OH-BTR, TTR, XTR, 5-Cl-1H-BTR, BTH, 2-OH-BTH, 2-MeS-BTH, 2-amino-BTH, 2-SCNMeS-BTH	1H-BTR , 1OH-BTR, TTR, XTR, 5-Cl-1H-BTR, BTH, 2-OH-BTH, 2-MeS-BTH, 2-amino-BTH, 2-SCNMeS-BTH	SPE: 2 mL deconjugated with β-glucuronidase (37 °C, 24 h) and extracted on Oasis HLB 6cc/200 mg with MeOH/ACN (1:1), dried and reconstituted in 200 μL MeOH/ACN (1:1)	UPLC: Waters Acquity Column: Waters Acquity BEH Shield RP18 (100 × 3 mm, 1.7 μm) Mobile phases: A (0.1% FA/W), B (MeOH/ACN, 1:1), flow 0.4 mL/min Gradient elution: 10% B, 0.5 min, 10–40% B, 0.5–1.0 min, 40–100% B, 1.0–2.0 min, 100% B, 2.0–6.0 min, 100–10%, 6.0–6.1 min, 10% B, 6.0–7.0 min	ESI(+)-MS2-MSM: Waters Xevo TQ-S Triple Quadrupole	0.005–0.51	Li et al. (2018b)
	1H-BTR , 5,6-MeMeBTR, 4-Me-BTR, 5-Me-BTR, 5-Cl-1H-BTR, BTH, 2-Me-BTH, 2-OH-BTH, 2-MeS-BTH, 2-amino-BTH, 2-SH-BTH	1H-BTR , 5,6-MeMeBTR, 4-Me-BTR, 5-Me-BTR, 5-Cl-1H-BTR, BTH, 2-Me-BTH, 2-OH-BTH, 2-MeS-BTH, 2-amino-BTH, 2-SH-BTH	SPME: to 1.33 mL urine 0.5 g NaCl added, diluted, extraction by 85 μm polyacrylate fibre, direct immersion, 40 min, room T. Thermal desorption at 290 °C for 10 min	GC: Thermo Fisher TSQ Quantum Column: Thermo TR-5MS (30 m × 0.25 mm i.d., 0.25 μm) Program: 65 °C, 1 min, 65–240 °C, 10 °C/min, 240–290 °C, 50 °C/min, 290 °C, 3 min	EI-MS2-MRM: Thermo Fisher Triple Quadrupole Quantum	4.9, 3.1, 1.8, 2.7, 4.9, 0.91, 0.80, 0.45, 0.006, 0.41, 3.11	Naccarato et al. (2014)
	SH-BTH	SH-BTH	Online SPE: 0.5 mL of urine deconjugated with β-glucuronidase (37 °C, overnight) and extracted on Waters Oasis HLB column (20 mm × 2.1 mm, 25 μm)	HPLC: Waters Alliance 2695 Column: Agilent Zorbax Eclipse XDB-C8 (50 mm × 4.6 mm, 5 μm) Mobile phases: A (W), B (1% FA/W), C (ACN), flow rate 0.2 mL/min Gradient elution: 70% A, 10% B, 20% C, 0–4 min, 70–0% A, 10% B, 20–90% C, 4–5 min, 0% A, 10% B, 90% C, 5–8 min, 0–70% A, 10% B, 90–20% C, 8–8.5 min, 70% A, 10% B, 20% C, 8.5–13 min	ESI(+)-MS2-MRM: Waters Quattro Ultima Triple Quadrupole	1	Gries et al. (2015)
Antimicrobials	MI, MCI	NMMA	LLE: 100 μL of urine freeze-dried and redissolved in 1 mL of ACN. Derivatized with PFBBr (K_2_CO_3_, 60 °C, 16h) and extracted with 2x 1 mL of HEX. Dried and reconstituted in MePh	GC: Agilent 7890 A Column: HP-5-MS (60 m × 0.25 mm i.d., 0.25 μm) Program: 90 °C, 1 min, 90–120 °C, 30 °C/min, 120 °C, 1min, 120–240 °C, 10 °C/min, 240–310 °C, 25 °C, 310 °C, 5 min	EI-MS2-MRM: Agilent 7000 Triple Quadrupole	0.5	Schettgen et al. (2017)
	MI, MCI	M-12	SPE: 500 μL of urine mixed with 500 μL of 100 mM ammonium formate buffer (pH 2.5). Addition of 10 μL formic acid and 10 μL of D3-M-12 (1 μg/mL) in water) as an internal standard, Injecting 100 μL of this solution into the LC/MS/MS system. Analyte enrichment and clean with Phenomenex Strata-X-column (20 × 2 mm; 20 μm) using water (pH 2.5) and methanol (90:10, v:v), flow rate of 0.5 mL/min. Backflush on the analytical column [Phenomenex C18(2), 150 × 4.6 mm, 3 μm, 100 A], separation from interferences using a gradient of water (pH 2.5) and acetonitrile	LC system: Agilent Technologies 1200 Infinity series), column: Phenomenex C18(2), 150 4.6 mm, 3 mm, 100 °, Mobile phases: A: water pH 2.5 (adjusted with formic acid), B: MeOH, C: ACN, flow rate 0.5 mL/min, column switching technique: 90% A for 5 min for analyte enrichment, valve switching to analytical column: 75% A, 25% C at flow rate 0.3 mL/min	Sciex API 5500 LC/MS/MS, ESI+, detection mode: MRM	0.2	Schettgen et al. (2021a,b)
Diisocyanates: MDI, 4TDI, 6TDI, NDI, PPDI, HDI	MDI	MDA	LLE: 2 mL of urine, deconjugated by sulphuric acid (100 °C, 90 min), neutralized, extracted with 4 mL DEE. Dried and reconstituted in 500 μL of MePh, derivatized by HFBA (55 °C, 60 min). Dried and reconstituted in 100 μL of MePh	GC: type not given Column: BP-5 (dimensions not reported, 1 μm) Program: 150 °C, 1 min, 150–280 °C, 10 °C/min, 280 °C, 1.5 min	NCI-SIM: type not reported	Not given	Mirmohammadi et al. (2013)
	MDI, 4TDI, 6TDI, NDI, PPDI	MDA, 4TDA, 6TDA, NDA, PPDA	SPE: 250 μL of urine deconjugated by HCl (80 °C, 4h), neutralized, extracted on Phenomenex Strata XC 30 mg/3mL with MeOH/IPA/NH_4_OH 75/20/5. Evaporated and reconstituted in 250 μL of mixed buffer	UPLC: Waters Acquity UPLC Column; ACE Excel2 SuperC18 (dimensions not reported) Mobile phases: A (100 mM Ammonium acetate/w), B (100 mM ammonium acetate/ACN, 5/95), flow: 0.5 mL/min Gradient elution: 10% B, 0–1 min, 10–30% B, 1–2 min, 30–90%, 2–2.5 min, 90–15% B, 2.5–4 min	APCI(+)-MS2-sMRM: Sciex 5500 Triple Quadrupole	0.03, 0.10, 0.10, 0.10, 0.33	(Bhandari et al. (2016)
	MDI, 4TDI, 6TDI	MDA, 4TDA, 6TDA	SPE: 250 μL of urine deconjugated by HCl (80 °C, 4h), neutralized, extracted with Phenomenex Strata XC 30 mg/3mL. Evaporated and reconstituted in 250 μL of ACN and derivatized with PBC (DMAP, 80 °C, 20 min), neutralized with HCl	HPLC: Agilent HP 1100 Column: Agilent SB C18 (4.6 × 150 mm, 3.5 μm), flow 1.0 mL/min Mobile phases: A (0.1% FA in 5% ACN/W), B (0.1% FA in ACN) Gradient elution: 70–90% B, 0–15 min, 90% B, 15–20 min	FLD: Agilent HP 1000, excitation 330 nm, emission 475 nm	0.00105–0.00163	Sun et al. (2018)
	HDI	TAHI	LLE: 1 mL of urine deconjugated with 100 μL sulphuric acid (100 °C, 16 h), neutralized and extracted with DCM. Derivatized with acetic anhydride (55 °C, 16 h). Dried and reconstituted in 200 μL of ACN (0.1% FA)	Nano-UPLC: Type not reported Column: Waters Symmetry C18 (100 mm × 100 μm, 3 μm) Mobile phases: A (0.1% FA/W), B (0.1% FA/ACN), flow: 0.6 μL/min Gradient elution: 95–10% A, 17 min	ESI(+)-MS2-MRM: Type not reported	0.03 (LOD)	Robbins et al. (2018)
	MDI	MDA	LLE: 190 μL of urine deconjugated with sulphuric acid (100 °C, 60 min) and neutralized and extracted with MePh. Dried and reconstituted in 200 μL of water	UPLC: Waters Acquity Column: Waters Acquity HSS T3 (50 mm × 2.1 mm, 1.8 μm) Mobile phases: A (0.1% ammonium acetate/W), B(0.1% ammonium acetate/MeOH), flow 0.6 mL/min Gradient elution: 5% B, 0.5 min, 5–90%, 3 min, 90%, 1 min	ESI(+)-MS2-MRM: Waters Xevo Triple Quadrupole	0.535	Lépine et al. (2019a)
	MDI	MDA	LLE: 1 mL of urine deconjugated with sulphuric acid (100 °C, 16 h), neutralized and extracted with MePh. Dried and derivatized by HFBA. Dried and reconstituted in EA.	GC: Agilent 6890 A Column: HP-5 (25 m × 0.32 mm × 0.17 μm) Program: 50 °C, 1 min, 50–145 °C, 10 °C/min, 145–165 °C, 5 °C/min, 165–300 °C, 25 °C/min, 300 °C, 10 min	NCI-MS-SIM: Agilent 5973N	0.1	Henriks-Eckerman et al. (2015)
	MDI, 4TDI, 6TDI, HDI	MDA, 4TDA, 6TDA, HDA	SPE: 190 μL of urine deconjugated with sulphuric acid (100 °C, 60 min) and neutralized and extracted with Waters MCX 30 mg/1 mL. Evaporated and reconstituted in borate buffer. Derivatized with acetic anhydride (instantly, room T).	UPLC: Waters Acquity Column: Waters Acquity HSS T3 (50 mm × 2.1 mm, 1.8 μm) Mobile phases: A (0.1% FA/W), B(0.1% FA/MeOH), flow 0.6 mL/min Gradient elution: 2% B, 1 min, 2–17% B, 3 min, 17–40% B, 1.5 min, 40–90%, 0.5 min, 90%, 1 min	ESI(+)-MS2-MRM: Waters Xevo Triple Quadrupole	1.33, 0.76, 1.24, 2.04	Lépine et al. (2020)
Pyrrolidones: NMP, NEP	NMP	5-HNMP, 2-NMSI	SPE: 600 μL urine extracted on Biotage Isolute ENV+ 100 mg/1ml, eluted with EA/MeOH (4:1). Evaporated and derivatized with MTBSTFA (110 °C, 60 min) and diluted with EA	Cooled injection GC: Agilent 7890 GC Column; DB-35MS (60 m × 0.25 mm i.d., 0.25 μm) Injector temperature program: 40 °C, 0.5 min, 40–240 °C, 120 °C/min, 240–260 °C, 600 °C/min, 600 °C, 10 min Column temperature program: 50 °C, 4 min, 50–90 °C, 25 °C/min, 90 °C, 1 min, 90–190 in, 190 min, 190–280 °C/min, 280 °C, 10 min	EI-MS: Agilent 5975	20, 5, 15, 5 (LOD)	Schindler et al. (2012)
	NEP	5-HNEP, 2-HESI					
	NMP	5-HNMP, 2-NMSI	Dilute and shoot: 100 μL of urine diluted 10-fold with water	HPLC: Agilent Column: Agilent Zorbax Eclipse Plus C18 (100 mm × 4.6 mm, 3.5 μm) Mobile phases: A (0.1% FA/W), B (ACN), 0.2 mL/min Gradient elution: 0–30% B, 0–9 min, 30–100% B, 9–9.5 min. 100% B, 9.5–10.5 min	ESI(+)-MS2-MRM:Agilent 6460 Triple Quadrupole	0.2	Haufroid et al. (2014); Suzuki et al. (2009)
	NMP	5-HNMP	Dilute and shoot: Urine diluted 10-fold with 5 mM ammonium formate buffer	UPLC: Waters Acquity I-Class Column; Waters HSS-PFP (100 mm × 2.1 mm, 1.8 μm) Mobile phases: A (5 mM ammonium formate/w), B (MeOH), flow 0.4 mL/min Gradient elution: 2.5% B, 0–0.6 min, 2.5–35% B, 0.6–2.8 min, 35–80%, 2.8–4.5 min, 80–2.5% B, 4.5–5.5 min	ESI(+)-MS-MRM: Sciex Triple Quadrupole 5500	0.274 (LOD)	Bhandari et al. (2019)
UV filters	MBC	MBC, CBC	SPE: 4 mL of deconjugated urine (β-glucuronidase, 37 °C, overnight) extracted on C18 (brand not reported) with acetone. Dried and reconstituted in W/MeOH/ACN (2:1:1)	UPLC: Waters Acquity Column: Waters Acquity BEH C18 (50 mm × 2.1 mm, 1.7 μm) Mobile phases: A (0.1% FA/w), B (0.1% FA/ACN/MeOH 1:1), flow 0.3 mL/min Gradient elution: 40% B, 0–1 min, 40–100% B, 1–1.1min, 100% B, 2 min	ESI(±)-MS2-MRM: Waters Acquity TQD Triple Quadrupole MBC esi +, CBC esi-	6, 6 (LOD)	León-González et al. (2013)
	MBC	MBC	SPE: 2 mL of deconjugated urine (β-glucuronidase, 37 °C, 12 h), centrifuged, diluted to 30 mL, extracted on Oasis HLB (200 mg, 6 mL) with EA. Dried and derivatized with BSTFA-TMCS (1%) at (room T, 30 m)	GC: Thermo Scientific TSQ Quantum XLS Column: RTX-5 (30 m × 0.25 mm, 0.25 μm) Program: 40–200 °C, 15 °C/min, 200–280 °C, 8 °C/min, 280–320°C, 10 °C/min	EI-MS2-MRM: Thermo Scientific TSQ Quantum XLS	3.454	Ao et al. (2018b)
	MBC	MBC	On-line	HPLC: Aria TLX-1 Columns: TurboFlow on TurboFlow Cyclone P (50 × 0.5 mm) and Hypersil Gold aQ columns (50 × 4 mm) Mobile phases: A, B, C (not reported), flow 0.7 mL/min Gradient elution: 100% A, 0–1.5 min, 41–32% A, 59–68% B, 2–5 min, 32–5% A, 68–95 B, 5–6 min, 100% C, 6–7.5 min, 0–5% A, 0–95% B, 7.5–8.5 min, 100% A, 8.5–9.5 min	APCI(+)-MS2-MRM: Thermo Scientific TSQ Triple Quadrupole	0.87 (LOD)	Krause et al. (2017); Frederiksen et al. (2017)
	MBC	MBC	2 mL of urine lyophilized, resuspended in 1 mL of 90% MeOH/W, centrifuged, and the Supernatant injected	HPLC: Type not reported Column: Sephasil Peptide C18 (250 mm × 4.60 mm, 5 μm) Isocratic elution: 88:12 MeOH/W, flow 0.5 mL/min	UV: SPD-6 UVD	2.9 ng/mL (LOD)	Janjua et al. (2008)
	MBC	CBC, CBC-OH	Online SPE: 0.5 mL urine deconjugated (β-glucuronidase, T and t not reported) and extracted on X Bridge C8 direct Connect HP (30 mm × 2.1 mm, 10 μm)	UPLC: Waters Acquity Column: Waters Acquity HHS C18 (150 mm × 2.1 mm, 1.8 μm) Mobile phases: Not reported Elution: Not reported	ESI (Polarity not reported)-MS2-MRM: Waters Xevo TSQ	0.15, 0.30	Leng and Gries, 2017)
Non-phthalate plasticizers Trimellitate	TEHTM	1-MEHTM, 2-MEHTM, 4-MEHTM, 5OH-1-MEHTM, 5OH-2-MEHTM, 5oxo-1-MEHTM, 5oxo-2-MEHTM, 5cx-1-MEPTM, 5cx-2-MEPTM, 2cx-2-MMHTM, 2cx-1-MMHTM	Online: 1 mL of urine deconjugated (β-glucuronidase, 37 °C, 2 h), centrifuged and injected on a restricted access material phase (Merck LiChrospher RP-18 ADS, 4 mm × 25 mm, 25 μm) with W/MeOH/FA 80/20/0.1 at 0.7 mL/min. Enrichment for 4 min	HPLC: Agilent 1100 Column: Restek Core-Shell Raptor Biphenyl (100 mm × 2.1 mm, 2.7 μm) Mobile phases: A (0.1% FA/W), B (0.1% FA/ACN), flow rate 0.3 mL/min Gradient elution: 0–2% b, 0–2 min, 2–35% B, 2–3 min, 35% B, 3–8 min, 35–55% B, 8–9 min, 55% B, 9–12 min, 55–80% B, 12–16 min, 80% B, 16–19 min, 80–2% B, 19–20 min, 2% B, 20–21 min	ESI(-)-MS2-MRM: Sciex API 200 Triple Quadrupole	4.6, 1.0, 0.7, 2.6, 1.5, 1.1, 2.6, 1.6, 2.6, 1.7, 2.4	Höllerer et al. (2018b)
	TEHTM	1-MEHTM, 2-MEHTM, 4-MEHTM	Online SPE: 50 μL of urine extracted on TurboFlow Cyclone column (50 mm × 0.5 mm) with 0.1% AA/W and 0.1% AA/ACN	UFLC: Shimadzu Prominence UFLC Column: Thermo Scientific Betasil phenyl/hexyl (100 mm × 3 mm, 3 μm) Mobile phases: A (0.1% AA/W), B (0.1% AA/ACN), flow 0.5 mL/min Gradient elution: 25% B, 0–5 min, 25–55% B, 5–9 min, 55–75% B, 9–14 min, 75–99% B, 14–15 min, 99% B, 15–24 min, 99–25% B, 24–24.1 min, 25% B, 24.1–25 min	ESI(-)-MS2-MRM: Sciex QTrap 5500	0.012, 0.044, 0.012	Pinguet et al. (2019)
	TEHTM	1-DEHTM, 2-DEHTM	SPE: 1 mL of deconjugated urine (β-glucuronidase, 37 °C, 90 min) extracted on Waters Oasis MAX 30 mg/3 mL with MeOH. Evaporated and reconstituted in ACN/W 1:1	HPLC: Agilent 1290 LC Column: Phenomenex Kinetex biphenyl RP (100 mm × 2.1 mm, 2.6 μm) Mobile phases: A (0.1% AA/W), B (0.1% AA/ACN), flow rate 0.2 mL/min Gradient elution: 15–45% B, 4 min, 15–100% B, 10 min, 100–15% B, 20 min, 15% B, 10 min	ESI(-)-MS-dMRM: Agilent 6460 Triple Quadrupole	0.10, 0.10	Bastiaensen et al. (2020); Been et al. (2019)
Adipates	DEHA	MEHA	Online SPE: 50 μL of urine extracted on TurboFlow Cyclone column (50 mm × 0.5 mm) with 0.1% AA/W and 0.1% AA/ACN	UFLC: Shimadzu Prominence UFLC Column: Thermo Scientific Betasil phenyl/hexyl (100 mm × 3 mm, 3 μm) Mobile phases: A (0.1% AA/W), B (0.1% AA/ACN), flow 0.5 mL/min Gradient elution: 25% B, 0–5 min, 25–55% B, 5–9 min, 55–75% B, 9–14 min, 75–99% B, 14–15 min, 99% B, 15–24 min, 99–25% B, 24–24.1 min, 25% B, 24.1–25 min	ESI(-)-MS2-MRM: Sciex QTrap 5500	0.044	Pinguet et al. (2019)
	DEHA	MEHA, OH-MEHA	SPE: 1 mL of deconjugated urine (β-glucuronidase, 37 °C, 90 min) extracted on Waters Oasis MAX 30 mg/3 mL with MeOH. Evaporated and reconstituted in ACN/W 1:1	HPLC: Agilent 1290 LC Column: Phenomenex Kinetex biphenyl RP (100 mm × 2.1 mm, 2.6 μm) Mobile phases: A (0.1% AA/W), B (0.1% AA/ACN), flow rate 0.2 mL/min Gradient elution: 15–45% B, 4 min, 15–100% B, 10 min, 100–15% B, 20 min, 15% B, 10 min	ESI(-)-MS-dMRM: Agilent 6460 Triple Quadrupole	0.15, 0.15	Bastiaensen et al. (2020); Been et al. (2019)
	DEHA	5OH-MEHA, 5oxo-MEHA, 5cx-MEPA	Online SPE: 300 μL of urine deconjugated (β-glucuronidase, 37 °C, 3 h) and extracted on Thermo Scientific TurboFlow Phenyl (50 mm × 0.5 mm)	HPLC: Agilent LC 1200 Column: Thermo Scientific Accucore Phenyl-X (150 × 3 mm, 2.6 μm) Mobile phases: A (0.05% AA/W), B (0.05% AA/ACN), flow rate 0.3 mL/min Gradient elution: 30% B, 0–2.5 min, 30–40% B, 2.5–4 min, 40–55% B, 4–19 min, 55–95% B, 19–20 min, 95% B, 20–22 min, 95–30% B, 22–23 min, 30% B, 23–27 min	ESI(-)-MS2-MRM: AB Sciex Qtrap 5500	0.05, 0.1, 0.05	Nehring et al. (2019)
	DnBA	MnBA, 3OH-MnBA, 3cx-MnPrA	Online SPE: 300 μL of urine deconjugated (β-glucuronidase, 37 °C, 2 h). Freezing and centrifuged. Samples were extracted on a Thermo Scientific TurboFlow Cyclone-P column (50 mm × 0.5 mm)	HPLC: Agilent 1260 Infinity II HPLC Column: Phenomenex Kinetex C18 (150 × 3 mm, 2.6 μm) Mobile phases: A (0.05% AA/W), B (0.05% AA/ACN), flow rate 0.4 mL/min Gradient elution: 12% B, 0–3.5 min, 12–45% B, 3.5–14 min, 45–95% B, 14–15 min, 95% B, 15–21 min, 95–12%, 21–21.5 min, 12% B, 21.5–29.5 min	ESI(-)-MS2-scheduled MRM: AB Sciex Triple Quadrupole 5500	0.1, 0.05, 0.5	Ringbeck et al. (2020)
Terephthalates	DEHTP	MEHTP	Online SPE: 50 μL of urine extracted on TurboFlow Cyclone column (50 mm × 0.5 mm) with 0.1% AA/W and 0.1% AA/ACN	UFLC: Shimadzu Prominence UFLC Column: Thermo Scientific Betasil phenyl/hexyl (100 mm × 3 mm, 3 μm) Mobile phases: A (0.1% AA/W), B (0.1% AA/ACN), flow 0.5 mL/min Gradient elution: 25% B, 0–5 min, 25–55% B, 5–9 min, 55–75% B, 9–14 min, 75–99% B, 14–15 min, 99% B, 15–24 min, 99–25% B, 24–24.1 min, 25% B, 24.1–25 min	ESI(-)-MS2-MRM: Sciex QTrap 5500	0.018,	Pinguet et al. (2019)
	DEHTP	MEHTP, OH-MEHTP	SPE: 1 mL of deconjugated urine (β-glucuronidase, 37 °C, 90 min) extracted on Waters Oasis MAX 30 mg/3 mL with MeOH. Evaporated and reconstituted in ACN/W 1:1	HPLC: Agilent 1290 LC Column: Phenomenex Kinetex biphenyl RP (100 mm × 2.1 mm, 2.6 μm) Mobile phases: A (0.1% AA/W), B (0.1% AA/ACN), flow rate 0.2 mL/min Gradient elution: 15–45% B, 4 min, 15–100% B, 10 min, 100–15% B, 20 min, 15% B, 10 min	ESI(-)-MS-dMRM: Agilent 6460 Triple Quadrupole	0.10, 0.10	Bastiaensen et al. (2020); Been et al. (2019)

To determine lysmeral and 7-HC biomarkers of exposure, two UPLC-based methods using ESI-MRM detection are presented (Pluym et al. 2016; Stoeckelhuber et al. 2017). Separation was achieved on a 100-mm C18 analytical column with a binary-solvent mixture. To detect biomarkers of exposure of lysmeral, methanol (MeOH), and an aqueous solution of 5 mM ammonium acetate with 0.025% ammonium hydroxide at a pH of 9.2 were used as mobile phases (Pluym et al. 2016). The method for the determination of 7-HCA included 0.1% formic acid (FA) in acetonitrile (ACN) and aqueous 0.1% FA. Both methods report low LOQs for the studied compounds (≤ 0.5 ng/mL); however, internal standards have to be custom synthesized (Pluym et al. 2016; Stoeckelhuber et al. 2017), which limits the possibilities for many laboratories.

### BTRs and BTHs

To determine benzotriazoles (BTRs) and benzothiazoles (BTHs), they are first deconjugated using β-glucuronidase and then extracted using LLE (Asimakopoulos et al. 2013a; Li et al. 2017; Zhou et al. 2018) or SPE (Asimakopoulos et al. 2013a; Li et al. 2018a) and online-SPE (Gries et al. 2015). Online-SPE requires the lowest sample volume (0.5 mL), whereas the presented LLE methods require between 0.6 and 1 mL of sample and the SPE methods between 1.3 and 2 mL. The most commonly used sorbent for SPE is mixed-mode Oasis HLB (Waters), and the separation method of choice is LC. The SPME method by Naccarato et al. (2014) yields the highest LOQs (0.4–4.9 ng/mL) of all the presented methods; however, it is also the only GC approach among the reported methods. Therefore, the high LOQ cannot be attributed to the sample preparation procedure alone, but also to the instrument of choice. The analytes were separated using a 30-m TR-5MS column and detected by MS in MRM mode. Although detecting BTRs and BTHs using GC is possible, the more complex sample preparation procedure and the relatively high LOQs than LC-based methods suggest that GC separation is less suitable for these compounds. The online-SPE method included only one compound, 2-mercapto benzothiazole (2SH-BTH) and achieved comparable LOQ with increased efficiency (time) and less sample handling compared to offline approaches (Gries et al. 2015). Despite other studies commercially obtaining isotopically labelled internal standards, the authors of this study custom synthesized the standards. The results do not seem to be impacted by this.

For BTRs and BTHs, four out of five methods (see Table 2) used a LC-ESI-MS2, but with different columns. Asimakopoulos et al. (2013a) used a 150-mm Zorbax SB aq column, whereas Zhou et al. (2018) and Li et al. (2017) used a 100-mm C18 column, Gries et al. (2015) used a 50-mm C8 and Li et al. (2018b) a BEH shield RP18 column, but in general, C18-based columns are the most commonly used and give optimal separation. All presented methods use a binary mixture of FA in water in the range of 0.1–1%, with all of the studies achieving comparably low LOQs (Table 2). The similarity in the separation and detection methods means it is most likely that the slight differences in achieved LOQs are attributed to the differences in sample preparation.

### Antimicrobials

Only two methods describe the determination of biomarkers of exposure of MI/MCI exposure in urine using both LLE (Schettgen et al. 2017) and SPE (Schettgen et al. 2021b). The LLE approach includes an additional derivatization step with pentafluorobenzyl bromide to increase lipophilicity. This step is omitted in the SPE procedure (Schettgen et al. 2017). However, the SPE method requires 0.5 mL of urine, whereas LLE yields better results (LOQ 0.5 ng/mL for NMMA) with 0.1 mL of sample, which is particularly important when sample volume is limited. The SPE method achieves an equally low LOQ (0.2 ng/mL); however, the analyte, in this case, is a different biomarker of exposure, a mercapturic acid metabolite of MI/MCI, named M12, which means that a direct comparison of the methods is not possible. However, both methods achieve low LOQs (Table 2), require a small sample volume and do not require complex procedures or installations in the laboratory. Important to note is that for NMMA isotopically labelled standards are commercially available, while for M-12, they have to be custom synthesized.

To determine MI/MCI biomarkers of exposure M-12, Schettgen et al. (2021a) used a LC-ESI-MRM with a 150-mm C18 column using a tertiary solvent system: water (pH 2.5, adjusted with FA), MeOH, and ACN. Together with SPE, the authors obtained a LOQ of 0.2 ng/mL, which is suitable for HBM studies (Table 2). Schettgen et al. (2017) presented a GC-based method with MRM detection for MI/MCI biomarkers of exposure NMMA based on a HP-5-MS 60m column. After LLE and derivatization with pentafluorobenzyl bromide (PFBBr) the method achieved a LOQ of 0.5 ng/mL (Table 2).

### Diisocyanates

In the case of diisocyanates, deconjugation is achieved using mineral acid hydrolysis. LLE is the most commonly applied method for extraction (Henriks-Eckerman et al. 2015; Lépine et al. 2019a; Mirmohammadi et al. 2013; Robbins et al. 2018) with SPE being used less often (Bhandari et al. 2016; Lépine et al. 2020; Sun et al., 2018) and cation exchange sorbents being the most used. Large differences in the required sample volume can be observed, as SPE methods require only a few hundred of microliters of the sample, while LLE requires larger volumes (1 and 2 mL). The exception is the method of Lépine et al. (2019a) where the authors use LLE with a volume of 250 μL. Only one method included a derivatization step with acetic anhydride (Bhandari et al. 2016; Lépine et al. 2019b, 2019a). By far, the lowest LOQ (0.001 ng/mL for MDA) was achieved by Sun et al. (2018) who applied SPE followed by derivatization with 4-(1-pyrene)butanoyl chloride. The data shows no difference in the LOQs obtained using LLE or SPE, albeit micro-SPE using a 96-well plate is more feasible for implementation in large-scale HBM schemes.

Most of the included LC methods involve UPLC, followed by HPLC, and nano-UPLC, with the C18 phase the most commonly used for separation. Three methods apply ESI-MRM detection (Lépine et al. 2020, 2019a; Robbins et al. 2018), whereas Sun et al. (2018) applied fluorescence detection (FLD). Bhandari et al. (2016) used atmospheric-pressure chemical ionization (APCI)(+)MRM detection. Three methods describe separation on a 100-mm or 150-mm C18 column (Bhandari et al. 2016; Robbins et al. 2018; Sun et al. 2018), whereas two studies used a 50-mm TSS T3 column (Lépine et al. 2020, 2019a). Sun et al. (2018) achieved the lowest LOQ (0.001 ng/mL) for MDA, but did require a derivatization step, which is omitted by Bhandari et al. (2016), while achieving a higher but equally satisfactory low LOQ as well (0.03 ng/mL) for MDA (Table 2). The method presented by Robbins et al. (2018) does not include MDA, but achieves a LOQ of 0.03 ng/mL for trisaminohexyl isocyanurate (TAHI). Internal standards for this compound are — in contrast to the other analytes presented here — not commercially available and have to be custom synthesized.

Henriks-Eckerman et al. (2015) and Mirmohammadi et al. (2013) used GC to determine MDA. Mirmohammadi et al. (2013) only briefly describe their method using negative chemical ionization (NCI) SIM detection and separation on a BP-5 column and do not report LOQ, whereas Henriks-Eckerman et al. (2015) achieved a LOQ of 0.1 ng/mL using a 25 m HP-5 column for separation and NCI-MS-SIM detection. Despite being suitable for HBM, the latter method requires a higher sample volume (1 mL) and derivatization with HFBA, leading to higher LOQs (Table 2) than the LC-based methods.

### Pyrrolidones

As a pre-treatment step, enzymatic deconjugation using β-glucuronidase is performed. The samples were either minimally prepared with the dilute-and-shoot approach using a 10-fold dilution with water (Bhandari et al. 2019; Haufroid et al. 2014; Suzuki et al. 2009) or extracted with SPE (Schindler et al. 2012). Based on the LOD/LOQ, required sample volume and overall sample preparation time, the dilute-and-shoot approach seems to yield better results. Only Schindler et al. (2012) included a derivatization step with *N*-methyl-*N*-tert-butyldimethylsilyl trifluoroacetamide (MTBSTFA) in their sample preparation to adapt the compounds for GC analysis. Applying derivatization, however, did not lead to lower LOQ. Isotopically labelled standards are commercially available for these analytes.

Two methods are presented to determine NMP biomarkers of exposure using LC-ESI-MRM: Haufroid et al. (2014) and Suzuki et al. (2009) achieved compound separation on a 100-mm C18 column using aqueous 0.1% FA and ACN as mobile phase. The second method separated compounds on a 100-mm HSS-PFP column using 5 mM aqueous ammonium formate and MeOH as mobile phase (Bhandari et al. 2019). Schindler et al. (2012) present a method for determining NMP and NEP using cooled injection GC and separation on a 60-m DB-35 MS column. Their method achieves LOQs (5–20 ng/mL) significantly higher than LC-based methods (Bhandari et al. 2019; Haufroid et al. 2014; Suzuki et al. 2009). The LOQs obtained using this method are too high for HBM, where the analytes are often present in trace concentrations (Table 1) and suggest that LC is a better approach for determining these compounds.

### MBC

Following enzymatic deconjugation, the most commonly applied sample preparation method for methylbenzylidene camphor (MBC) is offline SPE (Ao et al., 2018b; León-González et al. 2013), followed by online SPE (Frederiksen et al. 2017; Krause et al. 2017; Leng and Gries 2017). Janjua et al. (2008) used urine lyophilisation with reconstitution before analysis. Among those methods, online SPE requires the lowest sample volume (500 μL) for analysis, whereas other methods require a minimum of 2 mL. Additionally, the method presented by Leng and Gries (2017) yields the lowest LOQ (0.15 ng/mL for CBC), whereas a LOQ of 0.9 ng/mL (Frederiksen et al. 2017; Krause et al. 2017) is the second-lowest achieved showing that online-SPE is the most suitable sample preparation method for determining these compounds. Only one study (Ao et al. 2018b) included a derivatization step with N,O-bis(trimethylsilyl)trifluoroacetamide (BSTFA) for the instrumental analysis (GC), which did not increase the LOQ.

Four methods describe the detection of MBC and biomarkers of exposure of MBC using LC and, most commonly, a C18 phase. While most studies achieve separation on a 50–250-mm C18 column (Janjua et al. 2008; Leng and Gries 2017; León-González et al. 2013), Frederiksen et al. (2017) and Krause et al. (2017) used a 50-mm TurboFlow Cyclone P and a 50-mm hypersil Gold aQ column. Classical ESI-MRM detection is used by León-González et al. (2013) and Leng and Gries (2017), whereas Janjua et al. (2008) applied UV detection, specifically SPD-6 UVD, while Frederiksen et al. (2017) and Krause et al. (2017) chose APCI-MRM. Different mobile phase compositions are presented in the included studies. A binary mixture of aqueous 0.1% FA and 0.1% FA in ACN/MeOH (1:1) is used by León-González et al., while Frederiksen et al. (2017) and Krause et al. (2017) use a tertiary system without specifying the solvents. Isocratic elution with MeOH/water (88:12) is used in one study (Janjua et al., 2008), whereas Leng and Gries (2017) do not specify their mobile phases.

Krause et al. (2017) achieved by far the lowest LOQ for MBC (0.87 ng/mL), whereas the LOQs of the other studies range between 2.9 and 6 ng/mL for the same compound. Leng and Gries (2017) also achieved low LOQs, but for different biomarkers of exposure: CBC (0.15 ng/mL) and CBC-OH (0.3 ng/mL), which makes a comparison with other approaches difficult. Since Leng and Gries’s (2017) method involves time-saving online extraction with minimal sample handling and no derivatization, this method appears the most efficient one for the determination of MBC. Ao et al.’s (2018a) method for detecting MBC involves derivatisation with BSTFA+ trimethylchlorosilane (TMCS) followed by GC (30 m with TSQ Quantum XLS Column), with MS (MRM) detection. The obtained LOQ (3.5 ng/mL) is higher than that (0.9 ng/mL) obtained by Frederiksen et al. (2017) and Krause et al. (2017), making GC slightly less advantageous.

### Non-phthalate plasticizers

Following enzymatic deconjugation, extraction methods for biomarkers of exposure of TEHTM all involve SPE. The methods of Höllerer et al. (2018b) and Pinguet et al. (2019) include online-SPE, whereas Bastiaensen et al. (2020) and Been et al. (2019) describe an offline version. Only Pinguet et al. (2019) use 50 μL of the sample, whereas the other methods require 1 mL. In terms of LOQs, Pinguet et al.’s (2019) method gave the best results for mono-esters. In their method, Bastiaensen et al. (2020) and Been et al. (2019) determined di-esters at trace concentrations (LOQ 0.1 ng/mL). Although Höllerer et al.’s (2018b) method had the highest LOQ, they also analysed the highest number of analytes (*n* = 11) of interest in HBM studies. From this work, it is clear that online-SPE achieves the best results for determining TEHTM metabolites, although the inclusion of too many compounds can increase the LOQ by decreasing sensitivity, and researchers have to compromise between the advantages and disadvantages of each method based on their research question. Labelled standards are commercially available for TEHTM biomarkers of exposure.

Three online-SPE methods and one offline SPE method are published for determining DEHA biomarkers of exposure. None of the presented methods require a derivatization step for LC analysis. The offline SPE procedure (Bastiaensen et al. 2020; Been et al. 2019) requires a much higher sample volume compared to the online-SPE methods (1 mL vs 50–300 μL); however, it yields the highest LOQ (0.15 ng/mL for MEHA and OH-MEHA). The online-SPE methods by Nehring et al. (2019) and Pinguet et al. (2019) yield comparable results regarding LOQs. However, Pinguet et al. (2019) determined only MEHA, whereas Nehring et al. (2019) included OH-MEHA, oxo-MEHA, and cx-MEHA. Ringbeck et al. (2020) describe a method for the determination of DnBA biomarkers of exposure (MnBA, 3OH-MnBA, and cx-MnPrA) using online-SPE and report low LOQs (0.05–0.5 ng/mL). From this, it is evident that all of the presented methods are suitable for implementation in HBM studies, although Nehring et al.’s (2019) method has advantages in terms of preparation procedure (online), LOQ (comparable with others) and the number of analytes included (*n* = 3). While isotopically labelled internal standards are commercially available for DEHA transformation products, internal standards for DnBA biomarkers of exposure have to be custom synthesized.

For determining terephthalates, namely, DEHTP biomarkers of exposure, an online-SPE–based method (Pinguet et al. 2019) and an offline-SPE–based method (Bastiaensen et al. 2020; Been et al. 2019) were reported. The offline method requires a much larger sample (1 mL) than the online method (50 μL) and none of the procedures require derivatization. Although there are significant differences in sample preparation, the online method requires minimal sample handling compared to offline SPE. The benefits of this method are visible in terms of achieving lower LOQs; the online-SPE-based method achieved LOQ for MEHTP of 0.018 ng/mL, whereas the offline method achieved a LOQ of 0.1 ng/mL for the same analyte.

TEHTM biomarkers of exposure were determined exclusively with LC-based methods using ESI-MRM detection (Bastiaensen et al. 2020; Been et al. 2019; Höllerer et al. 2018b; Pinguet et al. 2019). While two of the methods chose a more traditional HPLC approach to determine biomarkers of exposure of TEHTM (Bastiaensen et al., 2020; Been et al. 2019; Höllerer et al. 2018b), Pinguet et al. (2019) utilized ultra-fast liquid chromatography (UFLC) instrumental setup for analysis. Additionally, differences between the methods lie in the choice of the analytical column and the mobile phase. Biphenyl columns are used in recent publications by Bastiaensen et al. (2020), Been et al. (2019), and Höllerer et al. (2018b), whereas Pinguet et al. (2019) utilize a phenyl hexyl column with a binary solvent system (0.1% acetic acid (AA)/water, 0.1% AA/ACN). The other studies also report using a binary system with different solvents. Höllerer et al. (2018b) achieved good separation using 0.1% FA in water and 0.1% FA in ACN, whereas Bastiaensen et al. (2020) and Been et al. (2019) used 0.1% AA in water and ACN. Among the methods, UFLC gave the best results in terms of LOQ (0.01–0.04 ng/mL) and required sample volume (50 μL). Bastiaensen et al. (2020) and Been et al. (2019) methods achieved promising results as well (LOQ 0.1 ng/mL), although they targeted different biomarkers of exposure. For the DEHTP biotransformation products included in this review (namely MEHTP and OH-MEHTP), isotopically labelled internal standards are commercially available.

For adipates, four LC-based methods are published, of which three target DEHA and one DnBA. All utilize LC-ESI-MRM, but Pinguet et al. (2019) used UFLC, whereas the other studies chose HPLC (Bastiaensen et al. 2020; Been et al. 2019; Nehring et al. 2019; Ringbeck et al., 2020). Three different types of columns were used, namely biphenyl (Bastiaensen et al. 2020; Been et al. 2019), phenyl hexyl (Pinguet et al., 2019), phenyl-X (Nehring et al. 2019), and C18 (Ringbeck et al. 2020). In all cases, the mobile consisted of a binary system of aqueous AA and AA in ACN, but Nehring et al. (2019) and Ringbeck et al. (2020) use 0.05% AA, whereas the other studies use 0.1% AA (Bastiaensen et al. 2020; Been et al. 2019; Pinguet et al. 2019). They all achieve comparable results for DEHA biomarkers of exposure (LOQs 0.04–0.15 ng/mL) and are suitable for HBM (Table 1). The lowest LOQ, however, was achieved with only one analyte (MEHA) in the method (Pinguet et al. 2019), which makes Nehring et al.’s (2019) method more appealing for implementation in HBM. The method for DnBA biomarkers of exposure also achieved low LOQs (0.05–0.5 ng/mL), making it suitable for HBM (Ringbeck et al. 2020).

Two studies determined DEHTP biomarkers of exposure. Pinguet et al. (2019) used a phenyl hexyl column and UFLC-ESI-MRM, while Bastiaensen et al. (2020) and Been et al. (2019) used a biphenyl column and HPLC-ESI-dMRM. Both studies use the same binary solvent system (aqueous 0.1% AA and 0.1% AA in ACN). Pinguet et al. (2019) achieved a lower LOQ for MEHTP (0.018 ng/mL), whereas 0.1 ng/mL was achieved by Bastiaensen et al. (2020) and Been et al. (2019). The latter also includes OH-MEHTP. A benefit to HBM of the UFLC approach is that it requires minimal sample (50 μL) and minimal sample handling (Pinguet et al. 2019).

## Conclusions

In this review, we reviewed six groups of compounds that have been overlooked in existing HBM schemes but are good candidates from the recent increase in their detection in urine and reports concerning their potential toxicity. This review summarizes the emerging techniques and current state-of-the-art analytical methods for inclusion in on-going HBM schemes. The methods were assessed in terms of their LOQs.

Looking at the analytical procedures, the most common first step in sample handling is enzymatic hydrolysis, which hydrolyses phase II metabolites to the parent compound or phase I metabolites. Accordingly, deconjugation is generally applied before analysis, except in the case of diisocyanates and nitro musks. Deconjugation was achieved using mineral acid hydrolysis for diisocyanates, while deconjugation was not applied for nitro musks.

Sample preparation varied widely within compound groups, but online-SPE stands out regarding the low sample volume required, limited sample handling, and achieved LOQs. However, the main drawback is the specific setup, which might not be feasible for every laboratory and offline SPE approaches were, therefore, the most commonly used. Despite being more time-consuming than online SPE and costlier than LLE, SPE offers high flexibility due to the variety of available SPE sorbents with broad polarity mixed-mode sorbents used most frequently, followed by ion-exchange sorbents for diisocyanates and certain phthalate alternatives. New techniques, such as solid-supported liquid-liquid extraction and ultrasound-assisted emulsification microextraction, represent new directions to achieve even lower detection limits and low sample volumes needed for HBM studies, as sample amount is limited, particularly for vulnerable populations, such as infants and children.

Compounds were almost exclusively analysed using separation techniques (GC and LC) hyphenated to mass spectrometry. Although both GC and LC can separate many compounds, LC-based approaches achieved better results in terms of detection limits. Due to the broader range of compound polarities, most studies chose LC. UPLC methods, achieving higher chromatographic resolutions than conventional HPLC and reduced analytical times, are in rapid development to fill the analytical gap, for example, in the case of lysmeral, 7-HC, BTRs, BTHs, and MBC. Furthermore, an even faster method is being developed, for example, UFLC, which was used to separate non-phthalate pesticides. It is also apparent that mass spectrometric detection is the most favoured in trace-level analyses in HBM, with a triple quadrupole analyser dominating the scene, followed by quadrupole-linear ion-trap (QTrap) analysers. To date, HRMS is scarcely used in the targeted analysis of these compounds. The most common ionization method was ESI with compound-dependent polarity followed by APCI, with the MS being operated almost exclusively in MRM mode ensuring high specificity and selectivity, which is important when dealing with complex samples like urine.

Various columns were used for GC separation depending on the compound type. Most studies used low polarity 5% diphenyl/95% dimethylsiloxane-based columns or equivalent for fragrances, MI and MCI, diisocyanates, and MBC, achieving the best separation of these compounds. Other column phases, such as non-polar 5% phenyl polysilphenylene-siloxane and mid polarity 35% phenyl/methylpolysiloxane were used for BTRs and BTHs and pyrrolidones, respectively. Like in the case of LC-based methods, the compounds were commonly detected using tandem MS in MRM mode, using GC-specific EI ionization and, less often, negative chemical ionization. Also, in the case of GC separation, derivatization was often applied to achieve sufficient volatility. Silylation forming trimethylsilyl ethers was most commonly used for reviewed compounds, followed by the formation of pentafluorobenzoyl and heptafluorobutyrate ethers and esters.

Most of the methods achieved LOQs in a ng/mL and sub ng/mL range; however, as data on exposure of broader populations are still scarce, actual levels might be lower and, thus, quantification is hindered. To improve the existing methodology, there remains a need for even more sensitive, specific, and accurate analytical methods that aid in comprehensive exposure assessment and inform health risk assessment. A necessary step is the lowering of LOQs and thus improving the sensitivities of existing methods to assess the exposure even at pg/mL levels.

The methods must also be fit-for-purpose and should comply with strict QA/QC procedures and report method validation procedures, such as accuracy, reproducibility, linearity, and matrix effects. Due to the complex nature of urine as a matrix it is essential to prepare matrix-matched calibration. A calibration matrix would be, in an ideal case, prepared by pooling different urine matrices to assure an average matrix effect. As urine samples vary by water content so does the dilution of matrix and, hence, the matrix effects which must be considered. This can be compensated by using isotopically labelled internal standards, although these are not yet available commercially for some compounds (namely selected biomarkers of exposure for: lysmeral, 7-HC, MI and MCI, and HDI). The suitability of the methods should be rigorously tested, not only by internal quality measures but also by establishing robust quality control systems and participating in external quality control protocols, such as EQUAS. The overview of the methods here shows that only one method reported the measurement uncertainty, indicating a general lack of comprehensive assessment and reporting of measurement uncertainty with the associated results in the exposure studies. Every sample processing and analytical protocol introduces a certain amount of measurement uncertainty through several laboratory steps and as every method differs in specific validation parameters, such as achieved LOD/LOQ and accuracy, so does the measurement uncertainty. Therefore, it is of paramount importance that it is reported along with the study results. Future development of certified reference materials for here presented compounds and production of certified calibration standards would enable a basis for traceability of results, which is fundamental for their comparability, particularly across different populations, as studied by HBM.

With advances in HRMS, the specificity and selectivity of targeted analysis could be improved even further as HRMS enables global-scale non-targeted analysis which informs on known and yet unknown chemicals present in urine. Identifying possible new metabolites and biomarkers of effect could further aid in understanding toxicity and the health risks associated with exposure to emerging contaminants. This review highlights several analytical techniques available to analyze these six groups of chemicals and should be applied to various populations to thoroughly assess the distribution and occurrence of the emerging contaminants discussed in this study in a large population. In addition, to improve cost- and time-efficiency, which is of immense importance in large cohort HBM studies, one comprehensive multi-residue method would be applied in an ideal case. However, with analysis of such a heterogeneous group of chemicals, this is impossible and compound prioritization in compliance with the research question should be performed and the most appropriate method applied. Nevertheless, to improve the existing knowledge of potential health risks associated with the exposure to the chemicals presented here, more studies into their biological effects are needed, especially when present in a real-life scenario, simultaneously, as a mixture.

## Data Availability

Not applicable.

## Abbreviations

*1-DEHTM*: 1,4-di-(2-ethylhexyl) trimellitate
*1H-BTR*: 1H-benzotriazole,
*1-MEHTM*: 1-mono-(2-ethylhexyl) trimellitate
*1OH-BTR*: 1-hydroxybenzotriazole
*2-amino-BTH*: 2-amino benzothiazole
*2cx-1-MMHTM*: 1-mono-(2-carboxymethylhexyl) trimellitate
*2cx-2-MMHTM*: 2-mono-(2-carboxymethylhexyl) trimellitate
*2cx-MMHTP*: Mono-(2-ethyl-2-carboxymethylhexyl) terephthalate
*2-DEHTM*: 2,4-di-(2-ethylhexyl) trimellitate
*2-HESI*: 2-hydroxy-*N*-ethylsuccinimine
*2-MEHTM*: 2-mono-(2-ethylhexyl) trimellitate
*2-MeS-BTH*: 2-methylthio benzothiazole
*2-NMSI*: 2-hydroxy-*N*-methylsuccinime
*2-OH-BTH*: 2-hydroxy benzothiazole
*2-SCNMeS-BTH*: 2-thiocyanomethylthio benzothiazole
*2-SH-BTH*: 2-mercapto benzothiazole
*3cx-MnPrA*: Mono-3-carboxy-n-propyl adipate
*3-NPA*: 3-nitrophthalic acid anhydride
*4-Me-1H-BTR*: 4-methyl-1H-benzotriazole
*4-MEHTM*: 4-mono-(2-ethylhexyl) trimellitate
*4TDA*: 2,4-toluenediamine, 4TDI – toluene 2,4 diisocyanate
*5,6-diMe-1H-BTr*: 5,6-dimethyl-1H-benzotriazole
*5-Cl-1H-BTR*: 5-chloro-1H-benzotriazole
*5cx-1-MEPTM*: 1-mono-(2-ethyl-carboxypentyl) trimellitate
*5cx-2-MEPTM*: 2-mono-(2-ethyl-carboxypentyl) trimellitate
*5cx-MEHTP*: 1-mono-(2-ethyl-5-carboxyhexyl) terephthalate
*5cx-MEPA*: mono-5-carboxy-2-ethylpentyl adipate
*5-HNEP*: 5-hydroxy-*N*-ethyl-2-pyrrolidone
*5-HNMP*: 5-hydroxy-*N*-methyl-2-pyrrolidone,
*5-Me-1H-BTR*: 5-methyl-1h-benzotriazole
*5OH-1-MEHTM*: 1-mono-(2-ethyl-5-hydroxyhexyl) trimellitate
*5OH-2-MEHTM*: 2-mono-(2-ethyl-5-hydroxyhexyl) trimellitate
*5OH-MEHA*: Mono-2-ethyl-5-hydroxyhexyl adipate
*5OH-MEHTP*: 1-mono-(2-ethyl-5-hydroxyhexyl) terephthalate
*5oxo-1-MEHTM*: 1-mono-(2-ethyl-5-oxohexyl) trimellitate
*5oxo-2-MEHTM*: 2-mono-(2-ethyl-5-oxohexyl) trimellitate
*5oxo-MEHA*: Mono-2-ethyl-5-oxohexyl adipate
*5oxo-MEHTP*: 1-mono-(2-ethyl-5-oxohexyl) terephthalate
*6TDA*: 2,6-toluenediamine
*6TDI*: Toluene 2,6 diisocyanate
*7-HC*: 7-hydroxycitronellal
*7-HCA*: 7-hydroxycitronellic acid
*AA*: Acetic acid
*ACN*: Acetonitrile
*ADBI*: Celestolide
*AHTN*: Tonalide
*AT*: Acetone
*APCI*: Atmospheric-pressure chemical ionization
*ATBC*: Acetyltributyl citrate
*ATII*: Traesolide
*BP*: Benzophenone
*BTH*: Benzothiazoles
*BSTFA*: N,O-Bis(trimethylsilyl)trifluoroacetamide
*BTR*: Benzotriazoles
*CBC*: 3-(4′-carboxybenzylidene) camphor
*DEE*: Diethyl ether
*DEHA*: Di-2-ethylhexyl adipate
*DEHTP*: Di-(2ethylhexyl) terephthalate
*DMAP*: 4-dimethylaminopyridine
*DMSO*: Dimethyl sulfoxide
*DnBA*: Di-*n*-butyl adipate
*FA*: Formic acid
*FD*: Fluorescence detection
*FR*: Flow rate
*HAD*: Hexamethylenediamine
*HDI*: 1,6-hexamethylene diisocyanate
*HESI*: Heated electrospray ionization
*HFBA*: Heptafluorobutyric anhydride
*HHCB*: Galaxolide
*IPA*: Isopropanol
*MA*: Musk ambrette
*MBC*: 3-(4-methylbenzylidene) camphor,
*MCI*: Methylchloroisothiazolinone
*MDA*: Methylene diphenyldianiline
*MDI*: Methylene diphenyl diisocyanate
*MEHA*: Mono-2-ethylhexyl adipate
*MEHTP*: Mono-(2ethylhexyl) terephthalate
*MI*: Methylisothiazolinone
*MK*: Musk ketone
*MnBPA*: Mono-*n*-butyl adipate
*MX*: Musk xylene
*NCI*: Negative chemical ionization
*NDA*: 1,5-naphthalenediamine
*NDI*: Naphthylen-1,5-diisocyanate
*NEP*: *N*-ethyl-2-pyrrolidone
*NMMA*: *N*-methylmalonamic acid
*NMP*: *N*-methyl-2-pyrrolidone
*PBC*: 4-(1-pyrene)butanoyl chloride
*PFBBr*: Pentafluorobenzyl bromide
*PLE*: Pressurized liquid extraction
*PP*: Polypropylene
*PPDA*: p-phenylenediamine
*PPDI*: 1,4-phenylene diisocyanate
*RT*: Room temperature
*SPE*: Solid phase extraction
*TAHI*: Trisaminohexyl isocyanurate
*TBBA*: Tert-butyl benzoic acid
*TBHA*: Tert-butyl hippuric acid
*TEHTM*: Tri-(2-ethylhexyl) trimellitate
*TMCS*: Trimethylchlorosilane
*TTR*: Tolyl benzotriazole
*UFLC*: Ultra-fast liquid chromatography
*XTR*: Xylyl benzotriazole
